# Climate-Driven Vegetation Distribution and Wetland Expansion at the Edge of Jiangjiadian Grassland, Northeastern China

**DOI:** 10.3390/plants14172785

**Published:** 2025-09-05

**Authors:** Xiaodong Wang, Xiaoqiang Li, Long Fei, Xiaohui Liu, Mei Zhang

**Affiliations:** 1 College of Geographical Sciences, Changchun Normal University, Changchun 130032, China; wangxd219@nenu.edu.cn (X.W.); lixiaoqiang@ccsfu.edu.cn (X.L.); feilong@ccsfu.edu.cn (L.F.); 2 Northeast Institute of Geography and Agroecology, Chinese Academy of Sciences, Changchun 130102, China; liuxh2752@126.com

**Keywords:** plant community dynamics, climate change, grassland edge, correlation analysis, structural equation

## Abstract

There is a close relationship between vegetation distribution and climate pattern in grassland areas, and offering insights into the climate–vegetation relationship may provide significant references for in-depth research on the response of plant community dynamics to climate change. In this study, we took the edge of the Jiangjiadian grassland in China as the research area. Using plant plots and climate data, the climate–vegetation relationship was revealed in relation to climate change on the grassland edge. The research results show that the relative frequency (RF), density (RD), height (RH), and coverage (RC) of *Phragmites australis*, a typical wetland plant, are the highest among the 10 common species tested. The path coefficient of mean temperature in October (MMTO) to the RD is 0.06 (*p* < 0.01), and the path coefficient of precipitation in October (POct) to the relative height (RH) is 0.62 (*p* < 0.05), indicating that the spatial pattern of climate has a significant impact on plant distribution. The temperature and the precipitation increases are associated with the trend regarding the transformation from grassland to wetland. Overall, 34 of the 360 correlation coefficients between climate indices and plant indices reached a significant level (*p* < 0.05), indicating that the relationship between wetland trends and the climate spatial pattern is very complex in relation to climate change in the past 25 years.

## 1. Introduction

The spatial distribution, species composition, and growth scenarios of grassland plants are connected with climate conditions [1]. Plant communities, in terms of community composition, growth status, and distribution in grasslands, are controlled by the spatial pattern of climate, and this is currently a hot research topic, with there being multiple research studies on the climate–vegetation relationship in certain regions [2]. Spatial patterns related to temperature and precipitation lead to the difference in grassland plant species’ composition under climate change [3]. In a previous study, it was found that spatial differentiation of precipitation causes the biodiversity index to increase in desert grassland plants, resulting in a greater variety of plant species in the typical grassland of Inner Mongolia, China [4]. Spatial differences in climate led to significant differences in plant species’ composition in the core area of the Inner Mongolia grassland, China [5]. Spatial heterogeneity of temperature and precipitation factors is largely responsible for the spatial diversity of β-diversity of alpine grasslands in the center of the Tibetan Plateau [6]. Reduced precipitation leads to increased spatial differences, which caused the suppression of some weed growth and a decline in the diversity of plant communities from 1981 to 2018 in the desert steppe of Yanchi, Ningxia, China [7]. However, in some grasslands around the world, there are no significant variations in species diversity as a result of climate spatial change [8]. Plant species richness varied insignificantly under heat stress effects such as drought (10 %), leading to greater spatial differences in the central region of the temperate grasslands of Europe from 1900 to 2022 [9]. In the heartland of the tallgrass prairie of the Flint Hills ecoregion of eastern Kansas, USA, grassland plants were found to be resistant to the droughts in 2011 and 2012, with no lasting changes in species composition [10]. One grass community in the center of the Montane grassland, Tasmania, Australia, was unaffected by large spatial differences in climate, as tested using 819 experimental observation data points [11]. As shown above, the relationships between the species composition of grassland plant communities and climate are different in different regions. Exploring the climate–vegetation relationship in sensitive areas, especially focusing on the edges of grassland areas, could provide references for further clarifying the mechanism of grassland plant variation.

While spatial differences in climate affect plant species, they also impact plant growth related to community variation. Grassland plants grew well in some places with high precipitation, but plants were hampered in another places where precipitation was insufficient. The spatial differences in precipitation amplified the spatial differences in the growth of grassland plants inside Norway’s grassland [12]. Adequate precipitation resulted in an average grassland coverage of 64% in the southern grassland, while low precipitation has limited the growth of grasslands in the northern grassland, with grassland coverage below 40%. The spatial pattern of precipitation led to significant spatial differentiation in the growth status of grassland plants at the innermost part of the Qinling Mountains in China [13]. Total coverage of plant communities in all places approached 100%, with the range of spatial distribution for temperature increasing from 0.24 to 3.41 °C over a period of 5 years (2002–2006) in the *Kobresia humilis* meadow in Qinghai China [14]. In humid environments, such as the core of the high-altitude places of grasslands of the Qinghai–Tibet Plateau, the dominant species of grassland plants, *Stipa sareptana*, has significantly reduced, while the secondary dominant species, *Carex alatauensis*, has significantly increased [15]. The relationships between plant growth of grassland communities and climate are different in different grasslands. Therefore, there is an urgent need for an in-depth analysis of the relationship between the plant growth of grassland communities and climate. For this study, we took the edge of the Jiangjiadian grassland in China as the study area, in which areas sensitive to climate change have been found previously, and aimed to study the relationship between plant growth and climate. We believe this study could provide a significant reference for further research on the impact of climate change.

The scientific question needs to adequately address whether there is a trend of grassland plant transformation driven by climate, whether the transformation direction of grassland is towards aridification (desertification) or humidification (wetland), and whether this transformation is related to the spatial pattern. Prior studies have addressed the climate–vegetation relationship under climate change, but the sampling points of these studies are concentrated in the core grassland areas. There is little research on the edges of grassland areas, which are more sensitive to plant change. Therefore, in this study, we analyzed the impact of the spatial variability of climate on the edges of grasslands in an agro-pastoral ecotone. The main contributions of this work are as follows:

(1) Analyzing the plant community characteristics of grassland edge areas using indices related to plant frequency, density, height, and coverage.
(2) Contributing to improving the understanding the differences in plant characteristics due to spatial differentiation of climatic conditions related to climate change.
(3) Revealing the climate–vegetation relationship via structural equations and correlation analysis. This study aims to offer insights into the spatial correspondence between climate and vegetation distribution, focusing on plant community dynamics, and provide a reference for future research on climate–vegetation relationships under climate change.

## 2. Materials and Methods

### 2.1. Study Area

**Figure 1:**
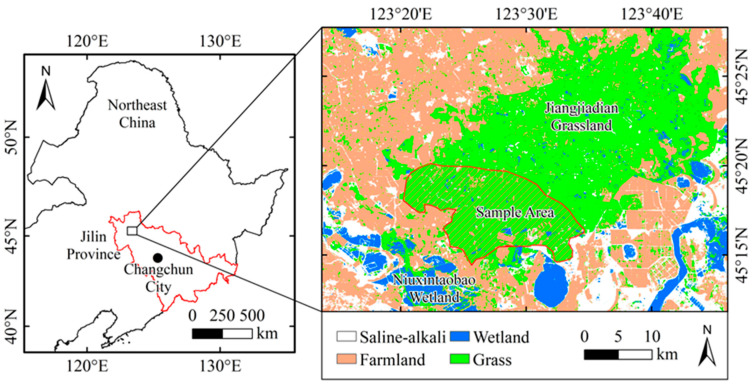
Study area and sample plot.

The study area is located at the edge of the Jiangjiadian grassland area in the west of northeastern China (123°18′–123°34′ E, 45°12′–45°22′ N), covering an area of 88 thousand km^2^, belonging to the transitional zone between humid and arid regions. The Jiangjiadian grassland is in the western part of the Songnen Plain, which has a temperate semi-humid and semi-arid climate [16]. The zonal vegetation here is temperate meadow grassland, which is the main grassland area in China [17]. The study area is an agricultural–pastoral ecotone with a temperate continental monsoon climate [18]. The winter is long and cold, while the summer is short and hot, with little wind and rain [19]. The annual mean temperature is 5.1 °C, and the annual total precipitation is 424 mm [20]. The Jiangjiadian grassland has a flat and open terrain and is one of the three natural *Leymus chinensis* grasslands in China [21]. The grassland vegetation mainly comprises *Leymus chinensis* plants, with coverage from 0.6 to 0.8, also including plants such as *Stipa bacailensis*, *Phragmites australis*, *Potentilla nivea*, *Chloris virgata*, *Artemisia anethifolia*, etc. [22]. The continuous decline in *Leymus chinensis* coverage has led to obvious signs of grassland degradation [23]. There are saline–alkali land and wetlands at the edge of the grassland area, and a small part of the land has been developed into farmland [24] (Figure 1). The grassland is adjacent to the Niuxin Taobao wetland, and under the influence of climate pattern, the wetland area has increased by 0.2 km^2^, with an expanding trend being evident [25]. The dominant species in the wetland area is *Phragmites australis*, with a coverage of 0.89. As the wetland area continues to expand, *Phragmites australis* has continued to invade the Jiangjiadian grassland [26]. The research area is located in a climate change-sensitive zone. In recent years, with spatial difference in climate increasing, the composition and growth trends of grassland plants have also undergone changes [27]. Therefore, the edge of the Jiangjiadian grassland is an ideal area for studying the climate–vegetation relationship.

### 2.2. Grassland Sampling

All 159 plots (each plot: 1 m × 1 m) were sampled from the edge of the grassland area from 1 to 10 July 2024. Based on the resolution (1 km) of grid points in climate data, each plot was set at the center point of each grid point on the grassland in the study area. The locations of all plots were located via GPS technology and then verified with the grid points in the climate data to ensure the accuracy of the sampling point location. The species, density, height, and coverage of all plants in each plot were recorded. The plant height was measured with a laser rangefinder using the pulse method [28], and plant coverage was measured using the line intercept method with a minimum gap size of 0.1 m [29]. The relative frequency (RF), density (RD), height (RH), and coverage (RC) were calculated for each plant in the plot, respectively. Plants with an RF value greater than 0.1 were deemed common plants. Using the coverage of *Leymus chinensis* in the typical grassland of Jiangjiadian as the standard [21], the plots with relative coverage of *Leymus chinensis* greater than 0.6 in the all plots were set as the components of the grassland community. Using the coverage of *Phragmites australis* in the typical wetland of western Jilin Province, China, as the standard [30,31], the plots with relative coverage of *Phragmites australis* greater than 0.5 in the all plots were set as the components of the wetland community. Each plot represented an area of 1 km^2^ (1 km × 1 km for each grid point), and the grassland community and wetland community areas were calculated separately to analyze grassland community variation.

### 2.3. Extraction and Analysis of Climate Spatial Data

**Figure 2:**
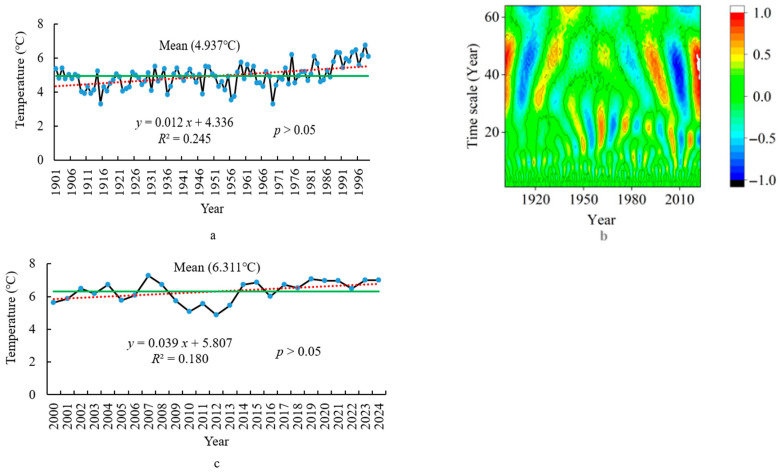
The AMT trend (**a**) (from 1901 to 1999), (**c**) (from 2000 to 2024) and wavelet analysis (**b**) (from 1901 to 2024) for the edge of the Jiangjiadian grassland.

**Figure 3:**
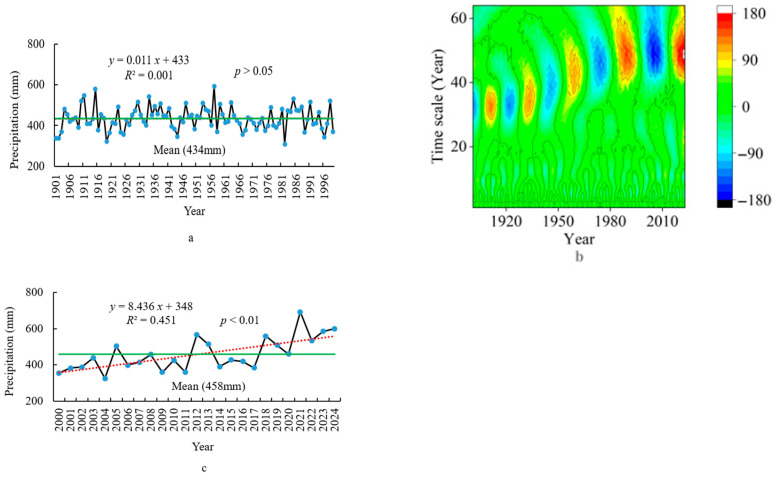
The ATP trend (**a**) (from 1901 to 1999), (**c**) (from 2000 to 2024) and wavelet analysis (**b**) (from 1901 to 2024) for the edge of the Jiangjiadian grassland.

**Figure 4:**
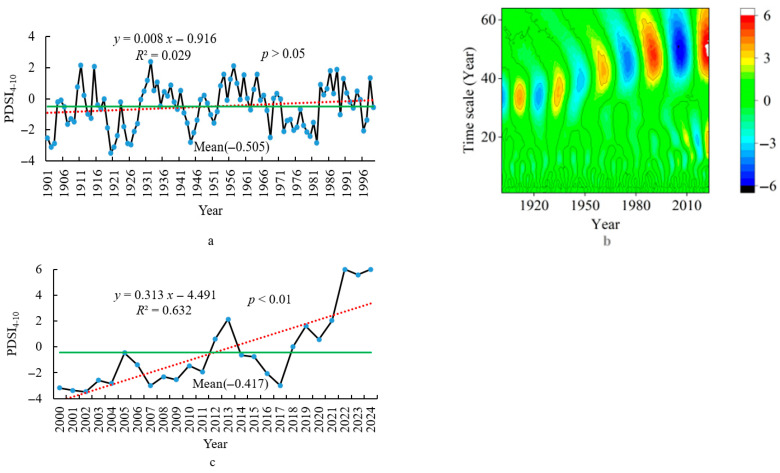
The dry–wet trend (**a**) (from 1901 to 1999), (**c**) (from 2000 to 2024) and wavelet analysis (**b**) (from 1901 to 2024) for the edge of the Jiangjiadian grassland.

Based on the method described by Fischer et al. (2020), the impact of climate on grassland plants is most significant over a 25-year time scale [32]. On the basis of this, the climate data was divided into different periods from 1901 to 2024 using wavelet analysis, and the period belonging to the past 25 years was extracted. By comparing the climate change trends and mean differences between 1901–1999 and 2000–2024, the characteristics of climate change in the past 25 years are revealed. At the same time, comparing the standard deviations of climate data from different grid points between 1901–1999 and 2000–2024 reveals the changes in climate spatial patterns over the past 25 years under climate change. This study was carried out using the National Tibetan Plateau Data Center Third Pole Environment Data Center, China (https://data.tpdc.ac.cn/en/data/71ab4677-b66c-4fd1-a004-b2a541c4d5bf accessed on 1 March 2024) from 1901 to 2024 (1 km resolution) [33]. Climate data for each grid point on the grassland study area were extracted separately, and the mean of all grid points on the grassland was calculated to give the climate data value in the study area. The mean of the climate data at 159 grid points for each year was calculated (1901–2024) on three climate indices (annual mean temperature (AMT), annual total precipitation (ATP), and the PDSI from April to October (growing season) (PDSI_4–10_)). Then, these three indices were divided into two periods, namely 1901–1999 and 2000–2024. Within each period, linear functions were performed with three climate indices as dependent variables and the year as an independent variable; next, the slope of each linear function was calculated separately (Figure 2, Figure 3 and Figure 4). Finally, by comparing the slopes of these linear functions at two periods, the climate characteristics in the past 25 years were understood and provided a basis for revealing changes in climate spatial patterns.

### 2.4. Data Analysis

Based on the method described by Fischer et al. (2020), the climate–vegetation relationship is most significant over a 25-year time scale [32]. Therefore, we used the mean of climate data from 2000 to 2024 for the study area. The climate data at each sampling point was extracted from grid points, and the mean of the climate data was calculated for 25 years at each separate grid point. Then, 14 ecological climate indices were obtained by computing the climate data for each grid point. The ecological climate indices used included temperature and accumulated temperature indices, namely AMT, ∑t ≥ 0 °C (growing season), ∑t ≥ 10 °C (active growth season), and monthly mean temperatures in January (MMTJ) (the coldest month), April (MMTA) (start of the plant growth stage), and October (MMTO) (end of the plant growth stage); precipitation indices, such as ATP, total precipitation from April to October (PAO) (growing season), total precipitation from July to August (PJA) (active growth season), precipitation in April (PApr), and precipitation in October (POct); and a dry–wet index, namely the humidity index (HI). The independent sample T-test found significant differences in climate indices among different grid plots (*p* < 0.001), and the spatial heterogeneity of climate was related to the differences in plant indices between different sampling points. Therefore, the 14 ecological climate indices and the 3 plant indices of common species (RD, RH, and RC) of the corresponding sampling points were assembled using structural equations (SEMs) (IBM SPSS company, Los Angeles, CA, USA) [34] and correlation analysis was carried out. The Kolmogorov–Smirnov test was used to test the normal distribution of all data, and finally, the 14 ecological climate indices and the 3 plant indices all passed the normal distribution test (*p* > 0.05). When constructing these SEMs, 14 climate indicators were used as climate latent variables, and 3 plant indicators were used as plant latent variables. All path coefficients of the SEMs were calculated using the linear mixed-effects models of the LME4 and SJSTATS packages and the two SEMS constructions were calculated using the PIECEWISESEM and LME4 packages in R software [35]. Finally, the two SEMS passed the significance test after 1000 iterations (*p* < 0.05). The correlation analysis was conducted using the correlation heatmap, that is, the correlation coefficients and significances were calculated using the ggthemes and ggcorrplot data packets in R software (R Core Team 4.1.2) (MathSoft company, Cambridge, MA, USA) [36].

## 3. Results

### 3.1. Plant Characteristics

#### 3.1.1. Common Plants

There are 10 species of all plants with an RF greater than 0.1 (Table 1). The typical wetland plant is *Phragmites australis* (PA), while the typical grassland plant is *Leymus chinensis* (LC). Plants co-occurring across the wetland and grassland plants include *Lolium perenne* (LP)*, Artemisia scoparia waldst* (ASW), *Cynanchum chinense* (CC), and *Eragrostis ferruginea* (EF). Salt–alkali-tolerant plants include *Stipa bacailensis* (SB)*, Iris lactea Pall* (I)*, Poa pratensis* (PP)*,* and *Panicum virgatum* (PV) (Table 1). The common plants include typical grassland, wetland, and salt–alkali-tolerant plants, which have a marginal effect across grasslands and wetlands. The RF is 0.69, the RD is 0.24, the RH is 0.19, and the RC is 0.23 for PA (Table 1), meaning it ranks first and has a significant advantage, indicating that the advantage of wetland plants is increasing and that grasslands are transforming into wetlands. The RF is 0.19 (ranked fourth), the RD is 0.14 (ranked second), the RH is 0.10 (ranked second), and the RC is 0.12 (ranked second) for LC (Table 1), indicating that LC still dominates the edges of grassland areas. The RD, RH, and RC values of the species co-occurring in wetland and grassland plants are relatively small (Table 1), indicating that the advantages of these plants are not obvious.

The RF of PA in the grassland community is 0.73 (Table 2), indicating that wetland plants have penetrated into typical grasslands, while the RF of LC in the wetland plants is 0 (Table 2), indicating that the wetland community has entered the grasslands. LP and ASW have a relatively high proportion in the grassland and wetland communities (Table 2), indicating that these two plants are growing as expected in both grassland and wetland communities.

#### 3.1.2. Species Relationship of Common Plants

Among the correlation coefficients (Rs) of the RDs of 10 common plants, only the R between the PA and the LC shows a significant negative correlation (R = −0.31, *p* < 0.01) (Table 3). The ecological niche differentiation increases and the degree of coexistence between PA and LC is relatively low. The Rs between the RDs of all plants reach the significant level (*p* < 0.05), except for the positive R (R = 0.20, *p* < 0.05) between ASW and CC (Table 3). The other Rs among the plants show significant negative correlations (*p* < 0.05), indicating that due to the increase in environmental gradients, niche differentiation leads to greater differentiation in plant growth heights. The significant negative correlations of RH between LC and the wetland plants, as well as other plants, indicates that the difference in growth height between typical grassland plants (LC) and other plants (including PA) is significantly increased. The R of the RC between PA and LP is 0.19 (*p* < 0.05), and the R of the RC between PA and LC is −0.30 (Table 3), indicating that the replacement of grassland plants by wetland plants is not the invasion of a single species, but rather the replacement of communities.

### 3.2. Climatic Characteristics

#### 3.2.1. Temperature Characteristics

There are nine high- and low-temperature periods, based on the wavelet analysis, with the last high-temperature period being 2000–2024 (Figure 2b). The mean of the AMT from 2000 to 2024 is 6.311 °C, which is 1.374 °C higher than that of the AMT from 1901 to 2024 (4.937 °C) (Figure 2a,c), indicating that the temperature is relatively high during the period when climate has an impact on vegetation change. The slope value (K = 0.039, *p* > 0.05) (Figure 2a) of the linear function between the AMT and the year from 2000 to 2024 is greater than the slope value (K = 0.012, *p* > 0.05) (Figure 2c) of the linear function between the AMT and the year from 1901 to 1999, indicating that the temperature increase is now faster than before. The standard deviation of the AMT among different grid points from 2000 to 2024 is 0.049 °C, which is 0.017 °C (0.032 °C) larger than that from 1901 to 2024, indicating that the rapidly increasing temperature leads to an increase in spatial differences in temperature among different grid points.

#### 3.2.2. Precipitation Characteristics

There are a total of nine high- and low-precipitation periods, with the last two periods in 2000–2024, based on the wavelet analysis (Figure 3b). The mean of the ATP from 2000 to 2024 is 458 mm, which is 24 mm higher than that of the ATP from 1901 to 2024 (434 mm) (Figure 3a,c), indicating that the precipitation is relatively high during the period when climate has an impact on vegetation change. The slope value (K = 8.436, *p* < 0.01) (Figure 3a) of the linear function between the ATP and the year from 2000 to 2024 is significantly higher than the slope value (K = 0.011, *p* > 0.05) (Figure 3c) of the linear function between the ATP and the year from 1901 to 1999, indicating that the increase in precipitation is now significantly faster. The standard deviation of the ATP among different grid points from 2000 to 2024 is 17 mm, which is 7 mm (10 mm) larger than that from 1901 to 2024, indicating that the rapidly increasing precipitation leads to an increase in spatial differences in precipitation among different grid points.

#### 3.2.3. Dry–Wet Characteristics

There are a total of nine high and low dry–wet periods, with the last two periods being especially significant (2000–2024), based on the wavelet analysis (Figure 4b). The PDSI_4–10_ from 2000 to 2024 is −0.417, which is 0.088 higher than the PDSI_4–10_ from 1901 to 2024 (−0.505) (Figure 2a,c), indicating that the humidity is high during the period when climate has an impact on vegetation change. The slope value (K = 0.313, *p* < 0.01) (Figure 4a) of the linear function between the PDSI_4–10_ and the year from 2000 to 2024 is significantly higher than the slope value (K = 0.008, *p* > 0.05) (Figure 4c) of the linear function between the PDSI_4–10_ and the year from 1901 to 1999, indicating that the humidification trend has been very significant recently. The standard deviation of the PDSI_4–10_ among different grid points from 2000 to 2024 is 0.17, which is 0.07 (0.10) larger than that from 1901 to 2024, indicating that the rapidly increasing humidity leads to an increase in spatial differences in humidification among different grid points.

### 3.3. Climate–Vegetation Relationship

#### 3.3.1. Temperature–Vegetation Relationship

**Figure 5:**
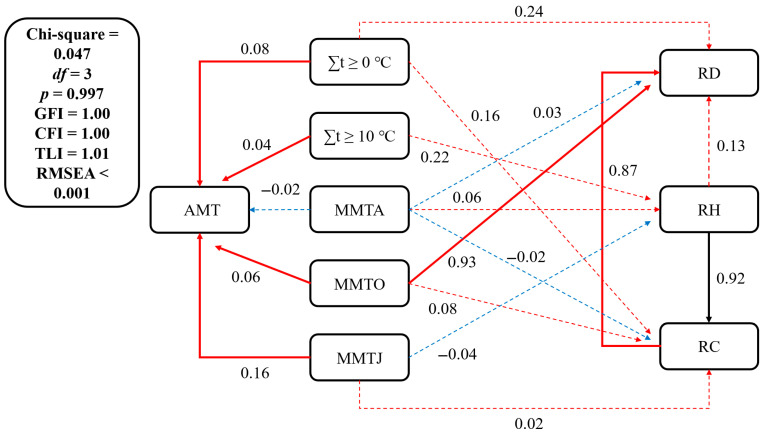
The structural equation of the temperature–vegetation relationship. The solid lines represent that the significant values of the path coefficients are less than 0.01, indicating that the relationship between temperature indices and plant factors is very significant in relation to the factors connected by solid lines. While the dashed lines represent that the significant values of the path coefficients are greater than 0.05, indicating that the relationship between temperature indices and plant factors is not significant in relation to the factors connected by dashed lines. AMT is annual mean temperature, MMTA is monthly mean temperature in April, MMTO is monthly mean temperature in October, MMTJ is monthly mean temperature in January, RD is relative density, RH is relative height, and RC is relative coverage.

**Figure 6:**
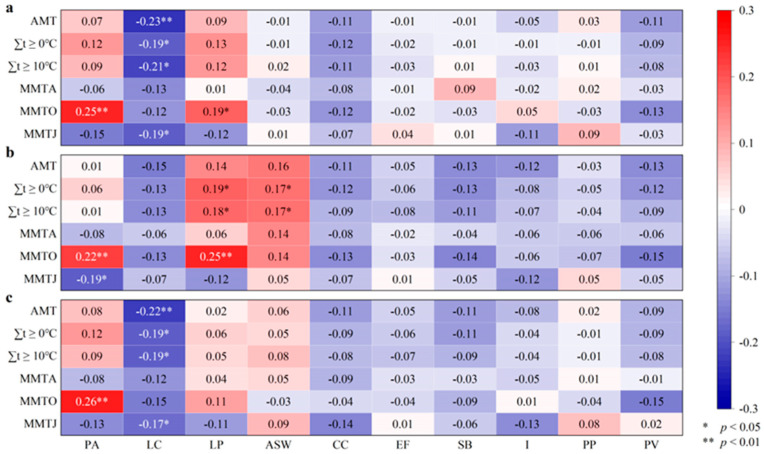
Heatmap of correlation coefficients between relative density (RD) (**a**), relative height (RH) (**b**), and relative coverage (RC) (**c**) of grasses and temperature indices. AMT is annual mean temperature, MMTA is monthly mean temperature in April, MMTO is monthly mean temperature in October, and MMTJ is monthly mean temperature in January, PA is *Phragmites australis*, LC is *Leymus chinensis*, LP is *Lolium perenne*, ASW is *Artemisia scoparia waldst*, CC is *Cynanchum chinense*, EF is *Eragrostis ferruginea*, SB is *Stipa bacailensis*, I is *Iris lactea Pall*, PP is *Poa pratensis*, and PV is *Panicum virgatum*.

The path coefficient of MMTO to the RD is 0.06 (*p* < 0.01) (Figure 5), indicating that the temperature and accumulated temperature have an impact on plant growth. The path coefficients of ∑t ≥ 0 °C, ∑t ≥ 10 °C, MMTO, and MMTJ to AMT are 0.08, 0.04, 0.16, and 0.06, respectively (*p* < 0.01) (Figure 5), indicating that the spatial distribution of these four temperature indicators reflects the spatial pattern of temperature. The path coefficients of the SEM show that the RH first significantly affects the RC (path coefficient of the RH to the RC: 0.92 (*p* < 0.01)), and then the RC significantly affects the RD (path coefficient of the RC to the RD: 0.87 (*p* < 0.01)) (Figure 5). The correlation coefficients (Rs) are all negative between the RH of the typical grassland plants (LC and the AMT) and ∑t ≥ 0 °C, ∑t ≥ 10 °C, MMTJ, MMTA, and MMTO (Figure 6b). The Rs are all significantly negative between the RD, the RC of LC and the AMT, ∑t ≥ 0 °C, ∑t ≥ 10 °C, MMTJ, MMTA, and MMTO (Figure 6a,c). According to the results of the SEM combined with correlation analysis, the growth heights of LC are inhibited at the sampling points corresponding to the grid points with relatively high temperatures, which leads to a decrease in coverage and density decrease. Therefore, the higher the temperature of the grid points, the greater the suppression of their growth height. The decreases in density through the inhibition of growth height eventually result in a decline in the growth of LC and grassland degradation in these points. The RH, RC, and RD of PA are positively correlated with the AMT, ∑t ≥ 0 °C, and ∑t ≥ 10 °C and significantly positively correlated with MMTO (Figure 6). The higher the temperature, especially at grid points with higher MMTO, the better the growth of PA at the sampling points corresponding to the grid points. The end of the growing season is the maturity period for plant seeds, and the high temperature may promote maturation and increase the number of seeds, which may increase the PA density. Therefore, high temperature may offer PA an advantage in rejecting LC as the dominant species at grid points with high temperatures. In addition, the Rs have reached significant positive correlation (*p* < 0.05) between LP and some temperature indices (Figure 6), indicating that the higher the temperature, the better the growth of coexisting plants at all the grid points or sampling plots.

#### 3.3.2. Precipitation–Vegetation Relationship

**Figure 7:**
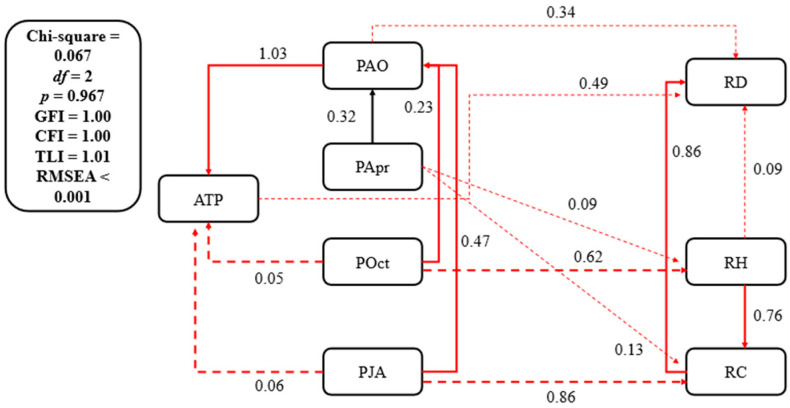
Structural equation of the precipitation–vegetation relationship. The solid lines represent that the significant values of the path coefficients are less than 0.01, indicating that the relationship between precipitation indices and plant factors is very significant in relation to the factors connected by solid lines. The short solid lines represent that the significant values of the path coefficients are less than 0.05, indicating that the relationship between precipitation indices and plant factors is significant in relation to the factors connected by short solid lines. The dashed lines represent that the significant values of the path coefficients are greater than 0.05, indicating that relationship between precipitation indices and plant factors is not significant in relation to the factors connected by dashed lines. ATP is annual total precipitation, PAO is total precipitation from April to October, PApr is precipitation in April, POct is precipitation in October, PJA is total precipitation from July to August, RD is relative density, RH is relative height, and RC is relative coverage.

**Figure 8:**
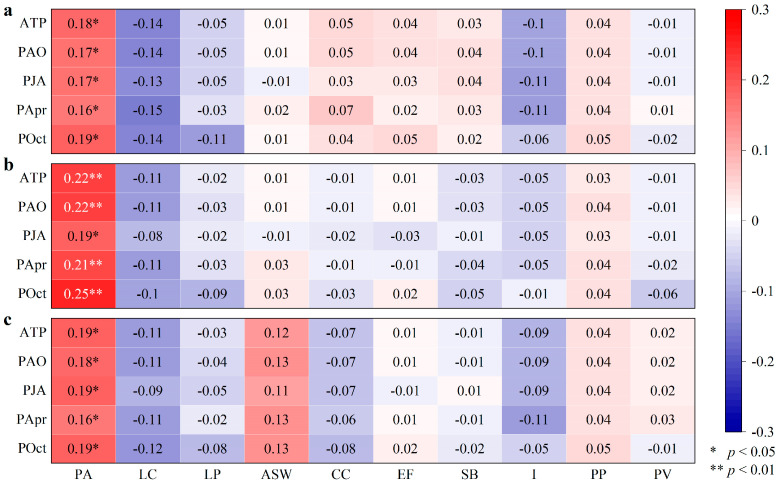
Heatmap of correlation coefficients between relative density (RD) (**a**), relative height (RH) (**b**), and relative coverage (RC) (**c**) of grasses and precipitation indices. ATP is annual total precipitation, PAO is total precipitation from April to October, PJA is total precipitation from July to August, PApr is precipitation in April, POct is precipitation in October, PA is *Phragmites australis*, LC is *Leymus chinensis*, LP is *Lolium perenne*, ASW is *Artemisia scoparia waldst*, CC is *Cynanchum chinense*, EF is *Eragrostis ferruginea*, SB is *Stipa bacailensis*, I is *Iris lactea Pall*, PP is *Poa pratensis*, and PV is *Panicum virgatum*.

The path coefficients of POct to RH and PJA to RC are 0.62 and 0.86, respectively (*p* < 0.05) (Figure 7), indicating that the precipitation pattern in the active growth period and the end of the growth season have a significant impact on the distribution of the growth of grassland plants. The path coefficients of POct and PJA to ATP are 0.05 and 0.06, respectively (*p* < 0.05) (Figure 7). The path coefficients of POct and PJA to PAO are 0.23 and 0.47, respectively (*p* < 0.01) (Figure 7). The path coefficient of PApr to PAO is 0.32 (*p* < 0.01), and the path coefficient of PAO to PJA is 1.03 (*p* < 0.01) (Figure 7). The results show that the spatial distribution of PJA is also reflected by the spatial pattern of PApr, POct, and PAO. The RD, RH, and RC of PA are significantly positively correlated with the ATP, PAO, PJA, PApr, and POct (*p* < 0.05) (Figure 8). According to the results of the SEM combined with correlation analysis, PA has a growth advantage, allowing it to become a dominant plant at the sampling points corresponding to the grid points with relatively high precipitation. The Rs between the RD, RH, and RC of LC and precipitation indices are negative, indicating that the more precipitation, the worse the growth of LC at all sampling points. However, all Rs did not reach a significant level (*p* > 0.05) (Figure 8), indicating that LC has the ability to resist the negative effects of high precipitation. The Rs between the RD, RH, and RC of some plants co-occurring in grasslands and wetlands and the precipitation indices are negative (as evidenced by the Rs between the RD, RH, and RC of LP and the precipitation indices (Figure 8)), indicating that high precipitation is detrimental to their growth at some points with high precipitation. Some plants have positive correlation coefficients (such as the Rs between the RD, RH, and RC of ASW and the precipitation indices (Figure 8)), indicating that high precipitation may promote their growth. Therefore, the spatial distribution of co-occurring plants is complex and closely related to spatial differences in precipitation.

#### 3.3.3. Dry–Wet Condition–Vegetation Relationship

**Figure 9:**
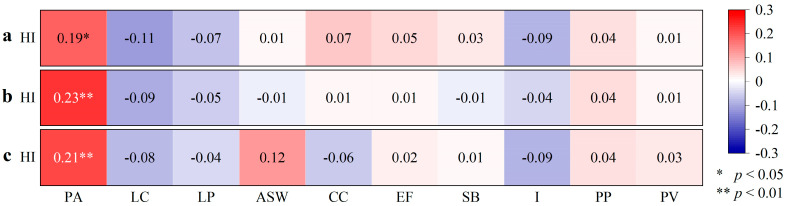
Heatmap of correlation coefficients between relative density (RD) (**a**), relative height (RH) (**b**), and relative coverage (RC) (**c**) of grasses and HI. HI is humidity index, PA is *Phragmites australis*, LC is *Leymus chinensis*, LP is *Lolium perenne*, ASW is *Artemisia scoparia waldst*, CC is *Cynanchum chinense*, EF is *Eragrostis ferruginea*, SB is *Stipa bacailensis*, I is *Iris lactea Pall*, PP is *Poa pratensis*, and PV is *Panicum virgatum*.

The Rs between the RD, RH, and RC of PA and HI are 0.19 (*p* < 0.05), 0.23, and 0.21 (*p* < 0.01) (Figure 9), respectively, indicating that the higher the humidity, the better the PA growth at some points with a high PDSI_4–10_. Therefore, humidity is a favorable condition for wetland expansion. The Rs between the RD, RH, and RC of LC and HI are negative and do not reach a significant level (*p* > 0.05) (Figure 9), indicating that the higher the humidity, the poorer the LC growth at some points with a high PDSI_4–10_. Therefore, humidification climate provides favorable factors for wetland plants to repel grassland plants.

## 4. Discussion

### 4.1. The Resistance of Grassland Plants

The results of the SEMs and correlation analysis indicate that wetland plants represented by PA are mainly distributed in sampling plots with high precipitation, high temperature, and relative humidity, which is consistent with the trend of climate change from 2000 to 2024. Previously, these plots were dominated by grassland plants, but now, due to climate change, wetland plants have invaded and become the main plants, indicating that climate change is closely related to grassland community dynamics. Only 34 out of 360 Rs between 10 common plants and all climate indices reached a significant level (*p* < 0.05) (Figure 6, Figure 8 and Figure 9), indicating that grassland plants do not passively respond to climate change, but have a certain degree of resistance to adverse climate conditions. The grassland plants have a certain self-regulation ability under the influence of climate change, in order to alleviate or eliminate adverse effects. Other studies have also found this phenomenon; for example, in the high-altitude grasslands of northwest China, the three main plants allocate more biomass underground to resist the adverse effects of accelerated evaporation and decreased soil moisture caused by rising temperatures [37]. In the Otindag Desert in China, from 2000 to 2019, a significant increase in dry–hot events caused a decline in vegetation loss, weakening the response to climatic extremes and increasing resistance [38]. In the Montane grassland and flooded savanna, the grassland exposure, sensitivity, and adaptive capacity significantly increased under the influence of precipitation and temperature changes, and disproportionately, the biodiversity of grassland enhanced the resilience of ecoregions to climate change [39]. The grassland ecosystem is not a passive response to climate change, but a positive response with a certain ability to seek benefits and avoid harm. Therefore, when studying the impact of climate change on ecosystems, it is important to pay attention to the ability to regulate climate change-induced effects.

### 4.2. The Salinization Phenomenon

SB, I, PP, and PV may be salt–alkali-tolerant plants (Table 1). In addition, in grassland communities, the salt–alkali plants are I and PV with an RF of 0.07 (Table 2), indicating that the presence of salt–alkali plants is relatively low in these communities. SB, I, and PP are not found in the grassland community, but they occupy a larger proportion of the wetland community’s area (Table 2), indicating that while wetland plants are expanding, there is also an increasing trend for salt–alkali-tolerant plants. Due to the semi-arid region in western Jilin, the phenomenon of salinization is more severe, and saline–alkaline soils have a long history in the region, so salt–alkali plants have become a common species in the region. PV has an RC value of 0.10 (ranked second), an RD value of 0.17 (ranked second), and an RH value of 0.24 (ranked fourth) (Table 2), which may indicate that salt–alkali plants coexist with LC in grassland communities. In wetland communities, the RF of PP is 0.29 (ranked second) (Table 2), indicating that salt–alkali plants occupy a relatively large position therein. PP has an RC of 0.14 (ranked third) and an RD of 0.52 (ranked first) (Table 2), meaning it may become a dominant companion species of PA. Under the condition of rising temperature, evaporation is vigorous [40]. The precipitation increases lead to an increase in local water content. Due to the closed terrain and poor drainage, the groundwater level rises, resulting in an increase in dissolved salts, and the salt in the water is carried to the surface and collected, leading to increased salinization [41,42]. As wetland plants gradually dominate, salt–alkali plants also show an expanding trend. Further verification is needed to determine whether the increase in salt–alkali plants is caused by climate change or whether it is the result of adaptive mechanisms developed over time. Overall, as a consequence of global warming, the ecological environment in semi-arid areas is deteriorating in many ways, including aridification, salinization, and wetland trends.

### 4.3. The Complexity of Climate–Vegetation Relationship

From the SEMs regarding the impact of temperature and accumulated temperature on plants community dynamics, only the path coefficient of MMTO on the RD reaches a significant level (*p* < 0.05) (Figure 5), the SEM of precipitation shows only the path coefficient of POct on the RH, and the path coefficient of PJA on the RC reaches a significant level (*p* < 0.05) (Figure 6). The results indicate that plant community dynamics do not accurately match local climate conditions, especially as vegetation does not immediately change with changes in climate conditions. Some factors, such as species competition [43], community succession [44], terrain [45], and soil [46], also play important roles in influencing the climate–vegetation relationship. In a study conducted in Inner Mongolia, specifically in the *Leymus chinensis* grassland, it was found that when the temperature rises to 0.086 °C/year, species competition intensifies, leading to *Stipa grandi* becoming the dominant species over *Leymus chinensi* [47]. Species competition makes the climate–vegetation relationships more complex under the background of climate change. The increase in precipitation accelerates the rate of succession of shrub communities to grassland in the desert–grassland junction of central Inner Mongolia [48]. Because the grassland aboveground biomass (AGB) increases with the rise in annual precipitation, the greater the terrain slope, the larger the grassland AGB in northern China [49]. In the Pacific Northwest, USA, increased soil nutrient availability caused the species richness of grassland to decline in the context of climate warming and drying [50]. The complexity of such responses provides favorable opportunities for human regulation [51], which may alleviate the negative impacts of climate change by regulating terrain and improving soil.

### 4.4. The Study’s Limitations

Due to the lack of plot data of grassland plants for continuous years, it is not possible to closely correlate plant data with climate data in time series, resulting in a lack of clarity on the response of plant dynamics to climate change over time. In the future, it is necessary to sample field surveys for continuous years to supplement plant data, and then plant data can be correlated with climate data to reveal the climate–vegetation relationship on continuous time series. At the same time, due to the influence of soil physical and chemical properties on plant growth, but without soil survey data in this study, there is no in-depth research on the role of soil in the climate–vegetation relationship. In addition, not all ecological factors influencing vegetation patterns are used to reveal the climate–vegetation relationship. Therefore, future research needs to further break through these limitations on the climate–vegetation relationship based on climate change.

## 5. Conclusions

Based on field vegetation and climate data, the characteristics of common plants were studied at the edge of the Jiangjiadian grassland, China. The recent climate change was analyzed over the past 25 years by comparing the climate characteristics from 2000 to 2024 which have an impact on plant community dynamics now with that from 1901 to 1999. Temperature and precipitation changes in the study area were analyzed using climate data from 1901 to 2024, and the climate–vegetation relationship related to spatial pattern of climate was revealed using SEMs and correlation analysis. (1) PA is ranked first among the 10 common plants studied, with the RF (0.69), RD (0.24), RH (0.19), and RC (0.23), indicating a wetland trend in the grassland. The RS of the RD (−0.31) and RH (−0.30) between PA and LC are significantly negatively correlated (*p* < 0.05), indicating that wetland gradually transforms grassland by repelling grassland plants. (2) The slope values of the linear trend functions of the AMT, ATP, PDSI_4–10_ and year from 2000 to 2024 are 0.027, 8.425, and 0.305 higher than those of 1901–1999, respectively. In the past 25 years (2000–2024), temperatures rose, precipitation increased, and there was significant humidification, which amplified the spatial heterogeneity of climate. (3) The path coefficients of MMTO on the RD of plants, POct on the RH of plants, and PJA on the RC have all reached a significant level (*p* < 0.05), indicating that the spatial distribution of plant growth is closely related to with the spatial differences in climate. The correlation analysis results show that wetland plants gradually replacing grassland plants is closely related to the spatial pattern of climate.

## Figures and Tables

**Table 1:** The statistics of 10 common plants. RF is relative frequency. RD is relative density. RH is relative height. RC is relative coverage. PA is *Phragmites australis*, LC is *Leymus chinensis*, L is *Lolium perenne*, ASW is *Artemisia scoparia waldst*, CC is *Cynanchum chinense*, EF is *Eragrostis ferruginea*, SB is *Stipa bacailensis*, I is *Iris lactea Pall*, PP is *Poa pratensis*, and PV is *Panicum virgatum*.

Plants	PA	LC	LP	ASW	CC	EF	SB	I	PP	PV
RF	0.69	0.19	0.43	0.31	0.14	0.11	0.13	0.11	0.11	0.11
RD	0.24	0.14	0.02	0.06	0.01	0.04	0.04	0.01	0.04	0.03
RH	0.19	0.1	0.04	0.07	0.01	0.03	0.04	0.02	0.04	0.04
RC	0.23	0.12	0.02	0.06	0.01	0.04	0.03	0.03	0.03	0.03

**Table 2:** The statistics of 10 common plants in both grassland and wetland communities. RF is relative frequency. RD is relative density. RH is relative height. RC is relative coverage. PA is *Phragmites australis*, LC is *Leymus chinensis*, LP is *Lolium perenne*, ASW is *Artemisia scoparia waldst*, CC is *Cynanchum chinense*, EF is *Eragrostis ferruginea*, SB is *Stipa bacailensis*, I is *Iris lactea Pall*, PP is *Poa pratensis*, and PV is *Panicum virgatum*. PT is the percentage of the community area relative to the total area, while PTP is the percentage of the community plot number relative to the total plot number.

CT	Plants	PA	LC	LP	ASW	CC	EF	SB	IP	PP	PV
GC	RF	0.73	1.00	0.13	0.47	0.27	0.00	0.00	0.07	0.00	0.07
	RD	0.11	0.74	0.02	0.10	0.05	0.00	0.00	0.03	0.00	0.17
	RH	0.29	0.38	0.27	0.13	0.07	0.00	0.00	0.04	0.00	0.24
	RC	0.10	0.76	0.03	0.10	0.05	0.00	0.00	0.02	0.00	0.10
WC	RF	1.00	0.00	0.24	0.29	0.06	0.12	0.18	0.00	0.29	0.12
	RD	0.69	0.00	0.12	0.24	0.01	0.10	0.07	0.00	0.15	0.04
	RH	0.35	0.00	0.39	0.15	0.05	0.39	0.44	0.00	0.52	0.39
	RC	0.74	0.00	0.18	0.13	0.02	0.08	0.10	0.00	0.14	0.08
	Area (km^2^)			PT (%)			Plot number (N)			PTP (%)	
GC	15			9.43			15			9.43	
WC	17			10.69			17			10.69	

**Table 3:** The significant correlation coefficients of three indices among all plants. * *p* < 0.05; ** *p* < 0.01. PA is *Phragmites australis*, LC is *Leymus chinensis*, LP is *Lolium perenne*, ASW is *Artemisia scoparia waldst*, CC is *Cynanchum chinense*, EF is *Eragrostis ferruginea*, SB is *Stipa bacailensis*, I is *Iris lactea Pall*, PP is *Poa pratensis*, and PV is *Panicum virgatum*.

	Significant Correlation Among Plants
RD	PA and LC (−0.31 **)
RH	PA and ASW (−0.20 *), PA and CC (−0.19 *), LC and LP (−0.19 *), ASW and CC (0.20 *), ASW and PV (−0.17 *), LC and PP (−0.21 **), LC and EF (−0.17 *)
RC	PA and LP (0.19 *), PA and LC (−0.30 **)

## Data Availability

Data are contained within the article.
